# Effectiveness of Oxycodone Hydrochloride (Strong Opioid) vs Combination Acetaminophen and Codeine (Mild Opioid) for Subacute Pain After Fractures Managed Surgically: A Randomized Clinical Trial

**DOI:** 10.1001/jamanetworkopen.2021.34988

**Published:** 2021-11-17

**Authors:** Deanne E. Jenkin, Justine M. Naylor, Joseph Descallar, Ian A. Harris

**Affiliations:** 1 University of New South Wales, South Western Sydney Clinical School, Sydney, Australia; 2 Whitlam Orthopaedic Research Centre, Liverpool, New South Wales, Australia; 3 Ingham Institute for Applied Medical Research, Liverpool, New South Wales, Australia; 4 Presently with Daffodil Centre, University of Sydney, a joint venture with Cancer Council New South Wales, Kings Cross, New South Wales, Australia; 5 Liverpool Hospital, South Western Sydney Local Health District, Sydney, New South Wales, Australia

## Abstract

**Importance:**

Patients with a surgically managed fracture are commonly discharged from the hospital with a strong opioid prescription, but limited evidence exists to support this practice.

**Objective:**

To test the hypothesis that strong opioids provide greater analgesia than mild opioids over the first week postdischarge from hospital after fracture surgical treatment.

**Design, Setting, and Participants:**

This double-blind, superiority, randomized clinical trial was conducted at a single-center, major trauma hospital in Sydney, Australia. Participants were inpatients who had sustained an acute nonpathological facture of a long bone or the pelvis, patella, calcaneus, or talus who were treated with surgical fixation and enrolled from July 27, 2016, to August 22, 2017. Data were analyzed from June through October 2018.

**Interventions:**

Initiation at discharge of oxycodone hydrochloride 5 mg of 10 mg (ie, 1 or 2 tablets) or combination acetaminophen and codeine 500 mg and 8 mg or 1000 mg and 16 mg (ie, 1 or 2 tablets) 4 times daily for a maximum duration of 3 weeks.

**Main Outcomes and Measures:**

The primary outcome was the mean of daily pain scores collected during week 1 of treatment measured using the Numerical Pain Rating Scale (NRS). Participants were asked to rate their mean pain over the previous 24 hours daily using an NRS score from 0 to 10, with 0 representing no pain and 10 representing the worst pain imaginable. The key secondary outcomes were EuroQol 5-Dimension 5-Level Questionnaire (EQ-5D-5L) responses, worst pain, medication adverse events, global perceived effect, and return to work.

**Results:**

A total of 120 patients with 1 or more acute orthopedic fractures requiring surgical fixation were randomized, including 59 patients in the strong-opioid group (43 [72.9%] men; mean [SD] age, 36.0 [14.1] years; mean oral morphine equivalent for days 1-7 of 32.9 mg) and 61 patients in the mild opioid group (47 [77.1%] men; mean [SD] age, 38.2 [13.5] years; mean oral morphine equivalent for days 1-7 of 5.5 mg). From days 1 to 7 postdischarge, the mean daily NRS mean pain score was 4.04 (95% Cl, 3.67 to 4.41) in the strong opioid group and 4.54 (95% Cl, 4.17 to 4.90) in the mild opioid group. The between-group difference of the primary outcome was not statistically significant (−0.50 [95% Cl, −1.11 to 0.12]; *P* = .11) despite a 6-fold increased dose of opioids being delivered in the strong opioid group.

**Conclusions and Relevance:**

This study found that treatment with strong opioid medication subacutely was not superior to treatment with milder medication for treatment of pain among patients with surgically managed orthopedic fractures. These findings suggest that ongoing first-line strong opioid use after discharge from the hospital should not be supported.

**Trial Registration:**

Australia New Zealand Clinical Trial Registry No.: ACTRN12616000941460

## Key Points

**Question:**

Does a strong opioid (oxycodone hydrochloride 5 mg or 10 mg 4 times daily) provide better analgesia than a mild opioid (acetaminophen codeine combination 500 mg and 8 mg or 1000 mg and 16 mg 4 times daily) when taken postdischarge among patients who underwent orthopedic surgical treatment?

**Findings:**

In this randomized clinical trial of 120 patients with 1 or more acute orthopedic fractures requiring surgical fixation, the mean of daily mean pain scores from day 1 to 7 postdischarge was 4.04 in the strong opioid group and 4.54 in the mild opioid group. The between-group difference was neither statistically nor clinically significant despite a 6-fold higher dose of opioid being delivered in the strong opioid group.

**Meaning:**

This study found that oxycodone did not provide superior pain relief compared with combination acetaminophen and codeine during the first 7 days of treatment despite a 6-fold higher dose of opioid being delivered in the strong opioid group.

## Teaser

This randomized clinical trial compares the effectiveness of a strong opioid (oxycodone hydrochloride) vs a mild opioid (acetaminophen codeine combination) when taken postdischarge for subacute pain among patients who underwent orthopedic surgical treatment for fracture.

## Introduction

Opioids have been used for centuries and are the most potent analgesic agents.^1,2^ Evidence supports the use of opioids to relieve moderate to severe pain, particularly acute and cancer pain.^3^ In these latter situations, the efficacy of opioids is extensively documented and broadly accepted. However, the evidence does not support using opioid therapy for chronic noncancer pain.^4^ The prescribing of opioids for chronic pain has increased despite evidence of the association of their long-term use with adverse outcomes.^5,6,7^While there is little debate over the short-term use of opioids, their use longer term is controversial, with increasing reluctance among some physicians to prescribe these medications.^8^ The most powerful opioid analgesics are also the most liable to be associated with abuse and addiction.^9^ With global attention on increased opioid prescription and the opioid abuse epidemic, reconsideration and reexamination of opioid pain management are being undertaken.

Orthopedic surgeons are among the top prescribers of opioid analgesics.^10^ Severe postoperative pain is common after orthopedic surgical treatment, and safe and effective management of this pain can be challenging for everyone on the health care team.^11^ Adequate relief of pain is a measure of patient satisfaction and may be associated with decreased chronic postsurgical pain; however, taking opioids for acute pain is associated with increased likelihood of long-term opioid use.^12^ Furthermore, increased initial opioid exposure (ie, increased total dose or longer-duration prescription) is associated with increased risks of long-term use, misuse, and overdose,^13,14^ suggesting that strong opioid prescription following discharge from the hospital should be considered carefully. Many choices are available for postoperative pain, but evidence to inform clinical choice after discharge in the subacute setting is sparse. However, patients with surgically managed fractures are commonly (and in many places, routinely) discharged home from the hospital with a strong opioid prescription.^15^

In the interest of minimizing the potential problems associated with long-term opioid use, we propose that discharge from the hospital signifies the most suitable time to transition people down the World Health Organization (WHO) analgesic ladder from step 3 (strong opioid) to step 2 (mild opioid) analgesics if strong opioids are not shown to be superior.^16^ With this in mind, we undertook a superiority trial to determine if a strong opioid (as per current practice) provided better analgesia than a mild opioid (a combination acetaminophen and low dose codeine) when taken postdischarge among patients who received orthopedic surgical treatment.

## Methods

### Study Design

This study is a double-blind, randomized clinical trial comparing oxycodone hydrochloride (strong opioid) vs combination acetaminophen and codeine (mild opioid) for the treatment of postdischarge pain after orthopedic fracture surgical treatment. This study and protocol (Supplement 1) was approved by the Hunter New England Health Human Research Ethics Committee, New South Wales, Australia. Participants provided written informed consent. This trial has been reported in line with the Consolidated Standards of Reporting Trials (CONSORT) reporting guideline recommendations for randomized clinical trials.

### Participants

Participants were patients admitted to 1 major trauma hospital in Sydney, Australia, with at least 1 acute fracture requiring surgical treatment from July 2016 to August 2017. The research team obtained relevant medical history to assess the patient against the eligibility criteria. Eligible participants were required to have sustained a nonpathological fracture of a long bone (ie, humerus, radius, ulna, femur, tibia, or fibula) or the pelvis, patella, calcaneus, or talus treated with surgical fixation; be age 18 years or older; and be able to comprehend the study protocol, written in English. Patients were excluded if they had known or suspected multisystem trauma injuries (eg, major head, chest, or abdominal injury); known or suspected major infection after surgical treatment; known or suspected opioid dependency; or contraindications to study treatment or were pregnant or breastfeeding.

### Randomization and Masking

A researcher not involved in participant recruitment or data collection generated a randomization schedule a priori using a computer-derived random number sequence. Study medication in the groups was identically overencapsulated and prepared according to the randomization schedule (concealed from study researchers and all participants) and sealed in medication-blinded blister packages. Upon the participant’s discharge from the hospital, the pharmacy dispensed a sealed medication package (in numerical order) to the participant. Once the package was opened, the participant was randomized to 1 of 2 groups (at a 1:1 ratio). The identical overencapsulated study packages ensured concealed allocation. Researchers, participants, and outcome assessors (DEJ and IAH) were blinded. To assess blinding integrity, the participants were asked to guess treatment allocation at the end of the study treatment period. Statistical analysis and preparation of the manuscript results and discussion (consisting of 2 versions, based on 2 possible group allocations) were performed prior to unblinding.

### Procedures

Before study enrollment, participants were administered oral oxycodone during their hospital admission and any other standardized hospital care. Members of the strong opioid group were prescribed oxycodone hydrochloride immediate release at 5 mg or 10 mg (ie, 1 or 2 tablets) 4 times per day. Members of the mild opioid group were prescribed acetaminophen and codeine at 500 mg and 8 mg or 1000 mg and 16 mg (ie, 1 or 2 tablets) 4 times per day. The maximum allowed daily dose (ie, 8 tablets/d) was supplied for 2 weeks (14 days). Study medication was titrated down to cessation during the third and final treatment week (days 15-21). Oxycodone monotherapy was selected to reflect standard therapy, while combination oxycodone and acetaminophen is not locally available. Combination codeine and acetaminophen was chosen as the comparator given that it was the lowest opioid dose available. If the participant considered pain well controlled during days 1 to 14, decreased dosing or cessation was allowed. Participants experiencing uncontrolled pain were discontinued from the study.

Data collection was conducted by researchers in person at baseline and via telephone at days 3, 7, 14, and 21 postdischarge. Participants recorded mean pain, worst pain, and adverse effects daily for 3 weeks or until study completion, with a minimum of days 1 through 7 collected, using a diary (eAppendix in Supplement 2). Study medication was returned at the end of the participants’ study period to calculate tablet use. Baseline data were collected on the hospital ward prior to discharge. Data were collected for the first 7 study days regardless of participants’ compliance with the study treatment.

### Primary Outcome

The primary outcome was pain measured by the Numerical Pain Rating Scale (NRS), which ranges from 0 to 10, with 0 representing no pain and 10 representing the worst pain imaginable. The primary outcome was collected daily, with participants asked to rate their mean pain over the previous 24 hours. The mean of the daily mean pain scores collected from day 1 to 7 postdischarge was calculated. The primary outcome was compared as between-group difference in daily pain during week 1 of treatment (ie, days 1-7).

### Secondary Outcomes

EuroQol 5-Dimension 5-Level Questionnaire (EQ-5D-5L) answers were collected at day 3 postdischarge and weeks 1, 2, and 3 postdischarge to assess health-related quality of life (HRQL). The EQ-5D-5L descriptive system is a preference-based HRQL measure with 1 question for 5 dimensions, including mobility, self-care, usual activities, pain and discomfort, and anxiety and depression.^17^ Worst pain was measured daily using the NRS. Participants were asked to rate their worst pain over the previous 24 hours on a scale of 0 to 10, with 0 representing no pain and 10 representing the worst pain imaginable. Medication adverse events were measured daily and recorded in the diary using a list of common adverse effects experienced, with participants asked to answer yes or no for each outcome (eAppendix in Supplement 2). The Global Perceived Effect was measured on days 7, 14, and 21; this questionnaire asks participants to compare their pain to what it was when their injury first occurred. It is measured on a Likert scale, from a score of −5 (vastly worse) to 0 (unchanged) to 5 (completely recovered). Return to work (with responses of yes [full or light duties] or no) was measured on days 7, 14, and 21, indicating a return to preinjury employment after injury.

### Other Data Collected

Descriptive data were collected on study entry during hospital admission. These data included age, sex, employment status, diagnoses, type of surgical treatment, mechanism of injury, insurance status, pain, and quality of life.

### Statistical Analysis

The primary comparison was the difference between the means of the daily mean pain scores over the first 7 days, measured on an 11-point NRS. Because group was a patient-level factor, sample size was estimated on this level. A sample of 46 participants per group (with 322 individual NRS pain scores) will provide more than 90% power to show at least a 1.0-unit (on an 11-point pain scale) advantage of oxycodone over acetaminophen and codeine assuming an SD of 1.5 between patients and a 1-sided type 1 error rate of 5%. A sample of 120 participants was randomized to allow for a loss to follow-up of 20%.

The distributions of characteristics of patients included in the study were described for the treatment groups. Significance testing to compare baseline covariates was not used. Data were analyzed by a statistician (JD) who was blinded. All analyses were intention-to-treat analyses (including outcomes for participants who discontinued the study drug). We conducted 3 additional per-protocol analyses of the primary outcome to compare participants who discontinued the study drug during day 1 to 7 for various reasons. Analysis 1 excluded participants who never commenced the allocated medication. Analysis 2 excluded participants who never commenced the allocated medication and those who discontinued the medication owing to adverse effects. Analysis 3 excluded participants who discontinued for any reason. Missing data did not meet the predetermined threshold for imputation. We had no missing data from baseline to primary end point (up to day 7). The primary analysis was a multilevel model using a random slope model with maximum likelihood estimation comparing the mean of daily mean pain scores between treatment groups while accounting for repeated measures over time by patients. The model included days and treatment groups as independent variables. The secondary outcomes were analyzed using similar multilevel models; student *t* test, and Fisher exact test were used, as appropriate. A generalized estimating equation with cumulative logit link was used to analyze EQ-5D-5L outcomes at days 3 and 7, clustered by patient with days (ie, 3 and 7), treatment group, and interaction days and treatment group as independent variables. The sample size was 1-sided, although there is no mechanism to allow for 1-sided *P* values in a multilevel model. For all analyses, a *P* value < .05 was considered statistically significant. The data analysis for this paper was generated using SAS Enterprise Guide statistical software version 7.15 (SAS Institute). Data were analyzed from June through October 2018.

## Results

During a 13-month period (July 27, 2016, to August 22, 2017), 899 patients were screened for participation. Of these, 161 individuals were eligible and 134 individuals provided written consent. A total of 120 patients were randomized at a 1:1 ratio, including 59 patients in the strong-opioid group (43 [72.9%] men; mean [SD] age, 36.0 [14.1] years) and 61 patients in the mild opioid group (47 [77.1%] men; mean [SD] age, 38.2 [13.5] years) (Table 1). Patients at baseline had a mean (SD) age of 37.1 (13.9) years, while 105 patients (87.5%) had sustained a single fracture and most fractures were of a lower extremity (67 among 120 patients randomized [55.8%]) (eTable 1 in Supplement 2). The variables appeared evenly distributed between treatment groups and between the randomized and nonrandomized samples (eTable 1 in Supplement 2). Baseline mean pain and worst pain intensity were initially moderate prior to hospital discharge (mean [SD] NRS mean pain score, 4.2 [1.56]; mean [SD] NRS worst pain score, 6.5 [1.9]). Participant flow through to the primary end point (ie, day 7) and beyond (ie, days 8-21) is shown in the Figure. There were 37 patients (30.8%), including 18 patients in the strong opioid group and 19 patients in the mild opioid group, who exited the trial after the primary end point (ie, beyond day 7).

**Table 1.** Baseline Characteristics of Participants^a^

Characteristic	Participants, No. (%)	
	Oxycodone (n = 59)	Acetaminophen and codeine (n = 61)
Sex		
Men	43 (72.9)	47 (77.1)
Women	16 (27.1)	14 (23.0)
Age, mean (SD), y	36.0 (14.1)	38.2 (13.5)
Height, mean (SD), cm	175.8 (10.6)	175.9 (9.5)
Weight, mean (SD), kg	85.1 (21.8)	89.9 (20.1)
BMI, mean (SD)^b^	27.4 (6.4)	29.0 (6)
Comorbidity ≥1	12 (20.3)	14 (23.0)
Education >secondary school	33 (56.0)	27 (44.3)
Recreational drug user	1 (1.7)	2 (3.3)
Weekly alcohol consumption	23 (39.0)	24 (39.4)
Premorbid pain medication use^c^	5 (8.5)	3 (4.9)
Nonsmoker^d^	47 (79.7)	40 (65.6)
No. of total fractures		
1	50 (84.8)	55 (90.2)
≥2	9 (15.2)	6 (9.8)
Mechanism of injury		
Road-related trauma	19 (32.2)	16 (26.2)
Fall	18 (30.5)	24 (39.4)
Blunt or crush trauma	21 (35.6)	20 (32.8)
Other	1 (1.7)	1 (1.6)
Region of fracture		
Upper extremity^e^	18 (30.6)	16 (26.2)
Lower extremity^f^	30 (50.8)	37 (60.7)
Pelvis	2 (3.4)	2 (3.3)
Multiple fracture regions	9 (15.2)	6 (9.8)
Insurance status		
Medicare	31 (52.5)	29 (47.5)
Private health insurance	23 (39.0)	22 (36.1)
Compulsory third party	2 (3.4)	3 (4.9)
Worker compensation	2 (3.4)	6 (9.9)
Other	1(1.7)	1 (1.6)
Employed	55 (93.2)	55 (90.2)
Length of stay, mean (SD), d	4.9 (4.9)	5.8 (5.3)
Admission to ICU	4 (6.8)	2 (3.9)
NRS score, mean (SD)		
Mean pain	4.0 (1.5)	4.4 (1.6)
Worst pain	6.3 (2.0)	6.7 (1.8)
EQ-5D-5L		
Mobility		
No problems	19 (32.2)	16 (26.2)
Problems^g^	40 (67.8)	45 (73.8)
Self-care		
No problems	4 (6.8)	4 (6.6)
Problems^g^	55 (93.2)	57 (93.4)
Usual activity		
No problems	0	1 (1.6)
Problems^g^	59 (100)	60 (98.4)
Pain		
No problems	2 (3.4)	1(1.6)
Problems^g^	57 (96.6)	60 (98.4)
Anxiety and depression		
No problems	41 (69.5)	35 (57.4)
Problems^g^	18 (30.5)	26 (42.6)
VAS score, mean (SD)	70.2 (12.6)	68.5 (13.0)

Abbreviations: BMI, body mass index; EQ-5D-5L, EuroQol 5-Dimension 5-Level Questionnaire; ICU, intensive care unit; NRS, numerical rating scale (range, 0-10); VAS, visual analogue scale (range, 0-100).

^a^ Baseline was collected during the hospital admission associated with index fracture.

^b^ BMI is calculated as weight in kilograms divided by height in meters squared.

^c^ Use of regular pain medication prior to admission.

^d^ Nonsmoker refers to individuals who did not smoke tobacco.

^e^ Includes humerus and radius or ulna.

^f^ Includes femur, patella, tibia or fibula, and calcaneus or talus.

^g^ Includes total of levels 2 (mild), 3 (moderate), 4 (severe), and 5 (extreme or unable).

**Figure.**
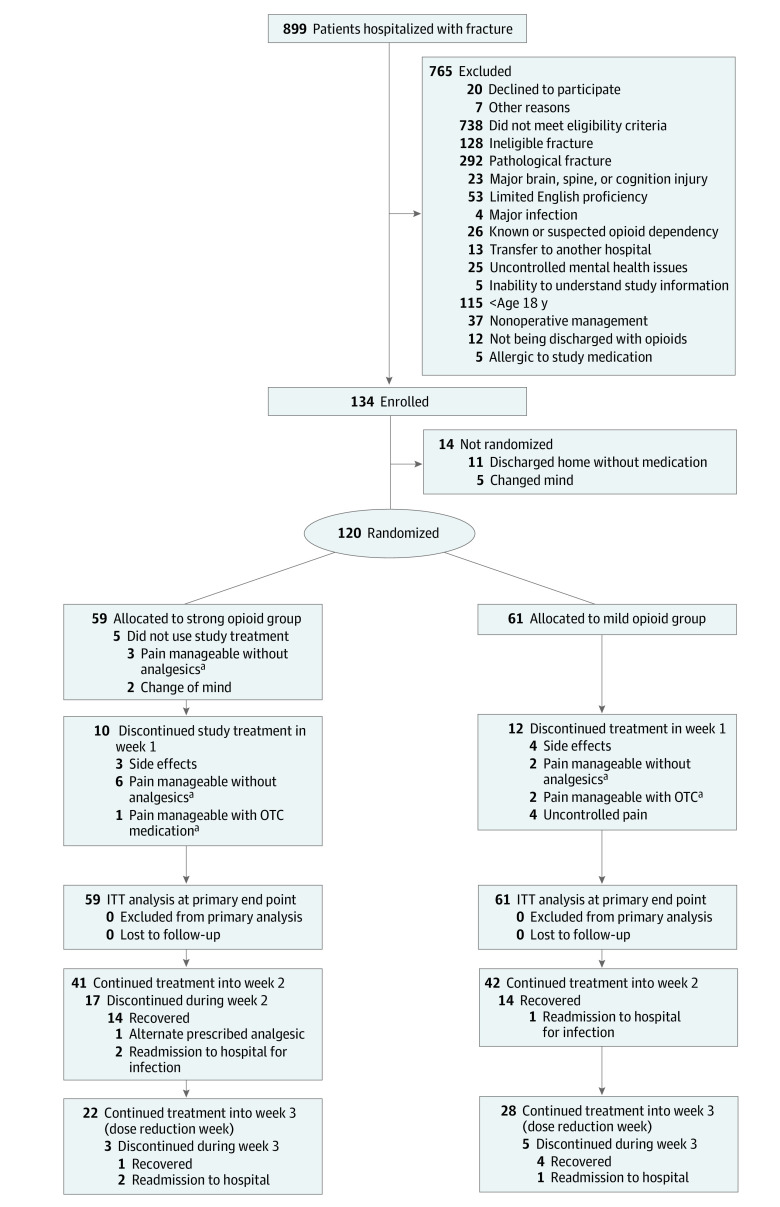
Flow of Participants Through Study Mild opioid indicates acetaminophen and codeine; OTC, over the counter; strong opioid, oxycodone hydrochloride. ^a^According to participant report.

From day 1 to day 7, the intraclass correlation coefficient (ICC) of daily NRS mean pain scores was 0.69. The mean of daily NRS mean pain scores was 4.04 (95% Cl, 3.67 to 4.41) in the strong opioid group and 4.54 (95% Cl, 4.17 to 4.9) in the mild opioid group. The difference between groups in the mean daily mean pain during days 1 to 7 after discharge from the hospital (ie, the primary outcome measure) was not statistically significant (−0.50 [95% Cl, −1.11 to 0.12]; *P* = .11). The per-protocol analyses supported the intention-to-treat primary analysis (Table 2). The mean (SD) number of adverse effects reported in the strong opioid group was increased vs the mild opioid group, but the difference was not statistically significant (Table 2). The number of patients reporting at least 1 adverse effect daily for the entire study period (days 1 to 21) is shown in eTable 2 in Supplement 2. Furthermore, no statistically significant difference was seen for any secondary outcomes.

**Table 2.** Outcomes at 1 to 21 d

Outcome	Total participants, No.		Mean (95% CI)^a^			*P* value
	Oxycodone	Acetaminophen and codeine	Oxycodone	Acetaminophen and codeine	Difference between groups	
**Primary outcome**						
Mean daily pain NRS score, days 1-7^b^						
Intention to treat	59	61	4.04 (3.67 to 4.41)	4.54 (4.17 to 4.90)	−0.50 (−1.11 to 0.12)	.11
Per protocol 1^c^	54	60	4.17 (3.72 to 4.62)	4.61 (4.19 to 5.04)	−0.44 (−1.05 to 0.17)	.16
Per protocol 2^d^	52	55	4.18 (3.74 to 4.62)	4.49 (4.06 to 4.91)	−0.30 (−0.91 to 0.30)	.32
Per protocol 3^e^	46	48	4.29 (3.83 to 4.75)	4.57 (4.12 to 5.02)	−0.28 (−0.92 to 0.35)	.38
**Secondary outcome**						
Mean pain NRS score, days 1-21^b^	59, 46, 21^f^	61, 46, 29^f^	3.17 (2.72 to 3.62)	3.62 (3.17 to 4.06)	−0.45 (−1.06 to 0.16)	.15
Worst pain NRS score^b^						
Days 1-7	59	61	5.77 (5.29 to 6.24)	6.06 (5.59 to 6.53)	−0.29 (−0.96 to 0.37)	.38
Days 1-21	59, 28, 15^f^	61, 35, 17^f^	4.50 (3.97 to 5.03)	4.80 (4.28 to 5.33)	−0.30 (−0.97 to 0.36)	.37
Mean daily tablet use^b^^,^^g^						
Days 1-7	59	61	4.36 (3.84 to 4.89)	4.59 (4.07 to 5.11)	−0.23 (−0.91 to 0.45)	.58
Days 1-21	59, 23, 12^f^	61, 25, 13^f^	3.33 (2.71 to 3.96)	3.49 (2.88 to 4.11)	−0.16 (−0.94 to 0.62)	.69
Mean daily adverse effects^h^						
Days 1-7	59	61	5.53 (4.22 to 6.83)	3.95 (2.80 to 5.10)	1.57 (−0.15 − 3.30)	.11
Days 1-21	59, 46, 21^f^	61, 46, 29^f^	8.68 (6.43 to 10.93)	5.66 (3.74 to 7.57)	3.02 (0.11 − 5.94)	.06
EQ-5D-5L VAS score, mean (SD)^h^						
Day 3	59	61	71.03 (11.56)	69.07 (15.04)	1.97 (−2.89 to 6.83)	.42
Day 7	59	61	74.68 (11.60)	73.92 (12.96)	0.76 (−3.71 to 5.23)	.74
Return to work, No (%)^i^						
Day 3	58	61	5 (8.62)	2 (3.28)	0.05	.26
Day 7	59	60	9 (16.36)	10 (18.18)	−0.01 (−0.15 to 0.12)	>.99
Day 14	40	41	8 (20.00)	10 (24.39)	−0.04 (−0.22 to 0.14)	.79
Day 21	25	28	6 (24.00)	8 (28.57)	−0.05 (−0.28 to 0.19)	.76
Global perceived effect, mean (SD)^h^						
Day 7	59	60	2.58 (0.87)	2.61 (0.83)	−0.02 (−0.33 to 0.28)	.88
Day 14	41	42	2.94 (0.87)	3.01 (0.78)	−0.07 (−0.43 to 0.29)	.69
Day 21	26	30	3.06 (0.87)	3.30 (0.81)	−0.24 (−0.69 to 0.21)	.29
Satisfaction, No (%)^i^	55	59	3.44 (0.79)	3.34 (0.78)	0.09 (−0.19 to 0.39)	.51
Complications, No. (%)^i^^,^^j^	59	61	7 (11.86)	2 (3.28)	0.09	.09

Abbreviations: EQ-5D-5L, EuroQol 5-Dimension 5-Level Questionnaire; NRS, numerical rating scale (range, 0-10); VAS, visual analogue scale (range, 0-100).

^a^ Values are presented as mean (95% Cl) using intention-to-treat analysis unless otherwise stated.

^b^ Analyzed using random slope models adjusting for time.

^c^ Excluded individuals who never commenced prescribed medication.

^d^ Excluded individuals as in protocol 1 and those who discontinued prescribed medication owing to adverse effects.

^e^ Excluded individuals as in protocols 1 and 2 and for all other reasons for discontinuation.

^f^ Sample size at days 7, 14, and 21, respectively.

^g^ Maximum of 8 tablets daily, measured on study exit.

^h^ Analyzed used 2-sample *t* test.

^i^ CIs are presented for statistical tests in which the normality assumption was satisfied. *P* values are from Fisher exact test.

^j^ Includes infection, reoperation, readmission, deep venous thrombosis, or pulmonary embolism.

Between-group daily mean tablet use was not statistically significantly different in the strong vs the mild opioid group as observed at days 1 to 7 (4.36 tablets [95% CI, 3.84 to 4.89 tablets] vs 4.59 tablets [95% CI, 4.07 to 5.11 tablets]; mean difference, −0.23 tablets [95% CI, −0.91 to 0.45 tablets]; *P* = .58) or days 1 to 21 (3.33 tablets [95% CI, 2.71 to 3.96 tablets] vs 3.49 tablets [95% CI, 2.88 to 4.11 tablets]; mean difference, −0.16 tablets [95% CI, −0.94 to 0.62 tablets]; *P* = .69). The morphine equivalent of the mean opioid use during day 1 to day 7 in the strong opioid group was 32.9 mg of oral morphine daily (21.96 mg oxycodone; conversion factor, 1.5), while the morphine equivalent of the mean opioid use in the mild opioid group was 5.5 mg of oral morphine daily (36.48 mg codeine; conversion factor 0.15).^18^ No secondary outcome measures, including patient-reported outcomes (eTable 3 in Supplement 2), were statistically significantly different between study groups.

At the end of study participation, among patients assigned to oxycodone, 8 patients (13.5%) correctly thought that they were taking oxycodone, 18 patients (30.5%) incorrectly thought that they were taking acetaminophen and codeine, and 33 patients (56.0%) would not guess an assignment. For participants assigned to combination acetaminophen and codeine, 21 patients (34.5%) correctly thought that they were taking combination acetaminophen and codeine, 16 patients (26.2%) incorrectly thought that they were taking oxycodone, and 24 patients (39.3%) could not guess an assignment.

## Discussion

Among patients discharged home following orthopedic surgical fixation for 1 or more acute traumatic bone fractures in this randomized clinical trial, oxycodone did not provide superior pain relief compared with combination acetaminophen and codeine during the first 7 days of treatment, despite a 6-fold higher dose of opioids being delivered in the strong opioid group. This study additionally found that oxycodone did not provide superior pain relief compared with combination acetaminophen and codeine over 3 weeks of treatment. The largest difference in mean NRS pain score between treatments (0.5) was not statistically significant and was less than 0.9 to 1.9, which is a commonly reported range to define the minimal clinically important difference (MCID) in pain.^19,20^ The 95% CIs of the primary intention-to-treat analysis included the prespecified conservative MCID selected, so the possibility of a clinically important difference (at 1.0 unit) cannot be excluded. However, the findings support the inference that there is no clinically important benefit to strong opioids vs mild opioids. The results further suggest that a mild opioid combination of acetaminophen and codeine may represent a viable alternative to the standard practice of prescribing a strong opioid analgesic for the treatment of surgically managed orthopedic fractures upon discharge.

Strong opioids are commonly prescribed at discharge after orthopedic fracture management.^21^ There is evidence that strong opioids do not provide superior pain relief to nonopioid or mild opioid alternatives within similar acute settings, but most studies providing this evidence are single-dose trials or of short duration.^22,23,24,25,26,27,28,29,30^ There is substantial evidence and acceptance that strong opioid use is not justified longer term in the treatment of chronic noncancer pain.^31,32^ Furthermore, a greater amount of initial opioid exposure (ie, increased total dose and a longer-duration prescription) is associated with increased risks of long-term use, misuse, and overdose.^13,14^ Considering the concern pertaining to opioid harms, dose reduction after acute hospitalization and after surgical treatment is a priority.

The concept of nonopioid and opioid dichotomy (ie, mild vs strong pain relief) providing varying levels of effectiveness is evident in the WHO pain ladder that has guided clinicians in the treatment of cancer since 1986 and noncancer pain at a later date.^16^ Depending on the intensity of pain, nonopioids (eg, ibuprofen and acetaminophen) are prescribed first and then mild opioids (eg, codeine) as necessary, followed by strong opioids (eg, hydrocodone and oxycodone). For individuals sustaining orthopedic fractures, initiation of strong opioids on admission is common. The findings of our study coupled with the existing literature suggest that a reduction down the WHO pain ladder from strong opioids to mild opioids upon discharge, rather than continuing strong opioids, should be supported. This change in prescribing habit could potentially help mitigate the harms from ongoing opioid use by decreasing the number of individuals initially exposed to opioids in the community and the subsequent risk of future addiction. In addition, this study challenges the concept of categorizing pain analgesics in general, at least in terms of their analgesic effects, given that single-dose trials of nonopioids, mild opioids, and strong opioids can provide similar analgesic effects in a variety of clinical settings.^33^ These findings suggest that current community perceptions and attitudes according to which strong opioids are considered the most effective and most appropriate approach in the management of subacute pain should be rethought.

### Strengths and Limitations

This study’s findings should be considered in light of its strengths and weaknesses. The study was adequately powered to detect a conservatively selected MCID (we used the smallest of the reported range within the study^18^) over the first week of treatment, group allocation was random, the loss to follow-up was minimal, analgesic consumption was closely monitored, and blinding was considered successful, given that most participants were unable to guess allocation. The study followed a pragmatic design, reflecting current prescribing habits, and limited the primary end point to the first week postdischarge assuming that many patients would recover and not require ongoing treatment after this point. This assumption was correct, with almost 31% of patients not requiring study treatment beyond day 7.

This study also has several limitations. Its single-center design and use of a hospital in a region with a high level of socioeconomic disadvantage may limit generalizability. The study excluded fragility fractures among older individuals; thus, the results do not apply to this patient population. The protocol allowed participant completion (dropout) after day 7, adding potential bias for findings from days 8 to 21. Additionally, we acknowledge that while we determined that strong opioids were not superior to mild opioids for pain relief, we could not determine if mild opioids were inferior. However, a larger sample size may have clarified whether adverse effects were increased in the strong opioid group, and if they were increased, establishing noninferiority may not be important.

## Conclusions

This study found that treatment with strong opioids was not superior to treatment with a milder opioid medication for postdischarge treatment of subacute pain among patients with surgically managed orthopedic fractures. These findings suggest that ongoing strong opioid use after discharge from the hospital should not be supported.

## Notes

Supplement 1.
Trial Protocol

Supplement 2. **eAppendix.** Extract From Participant Daily Diary **eTable 1.** Baseline Characteristics for Participants Giving Consent: Randomized vs Nonrandomized **eTable 2.** Reported Daily Adverse Effects **eTable 3.** Quality of Life Scores for EuroQol 5-Dimension 5-Level Questionnaire Dimensions at Days 3 and 7

Supplement 3.
Data Sharing Statement

## References

[1] Fields HL. The doctor’s dilemma: opiate analgesics and chronic pain. Neuron. 2011;69(4):591-594. doi:10.1016/j.neuron.2011.02.001 21338871 PMC3073133

[2] Pasternak GW, ed. The Opiate Receptors. Humana Press/Springer; 2011. The Receptors. doi:10.1007/978-1-60761-993-2

[3] Macintyre P, Schug S, Scott D, Visser E, Walker S, eds; APM: SE Working Group of the Australian and New Zealand College of Anaesthetists and Faculty of Pain Medicine. Acute Pain Management: Scientific Evidence (3rd Edition). Australian and New Zealand College of Anaesthetists Faculty of Pain Medicine; 2010. Accessed September 27, 2021. https://www.apsoc.org.au/PDF/Publications/Acute_pain_management_-_scientific_evidence_-_third_edition.pdf

[4] Els C, Jackson TD, Hagtvedt R, . High-dose opioids for chronic non-cancer pain: an overview of Cochrane Reviews. Cochrane Database Syst Rev. 2017;10(10):CD012299. doi:10.1002/14651858.CD012299.pub2 29084358 PMC6485814

[5] Compton WM, Volkow ND. Major increases in opioid analgesic abuse in the United States: concerns and strategies. Drug Alcohol Depend. 2006;81(2):103-107. doi:10.1016/j.drugalcdep.2005.05.009 16023304

[6] Dhalla IA, Mamdani MM, Sivilotti ML, Kopp A, Qureshi O, Juurlink DN. Prescribing of opioid analgesics and related mortality before and after the introduction of long-acting oxycodone. CMAJ. 2009;181(12):891-896. doi:10.1503/cmaj.090784 19969578 PMC2789126

[7] Leong M, Murnion B, Haber PS. Examination of opioid prescribing in Australia from 1992 to 2007. Intern Med J. 2009;39(10):676-681. doi:10.1111/j.1445-5994.2009.01982.x 19460051

[8] Blake H, Leighton P, van der Walt G, Ravenscroft A. Prescribing opioid analgesics for chronic non-malignant pain in general practice - a survey of attitudes and practice. Br J Pain. 2015;9(4):225-232. doi:10.1177/2049463715579284 26526705 PMC4616983

[9] Coyle DT, Pratt C-Y, Ocran-Appiah J, Secora A, Kornegay C, Staffa J. Opioid analgesic dose and the risk of misuse, overdose, and death: a narrative review. Pharmacoepidemiol Drug Saf. 2018;27(5):464-472. doi:10.1002/pds.4366 29243305

[10] Volkow ND, McLellan TA, Cotto JH, Karithanom M, Weiss SR. Characteristics of opioid prescriptions in 2009. JAMA. 2011;305(13):1299-1301. doi:10.1001/jama.2011.401 21467282 PMC3187622

[11] Pasero C, McCaffery M. Orthopaedic postoperative pain management. J Perianesth Nurs. 2007;22(3):160-172. doi:10.1016/j.jopan.2007.02.004 17543801

[12] Pino CA, Wakeman SE. Prescription of opioids for acute pain in opioid naïve patients. UpToDate. Accessed May 16, 2019. https://www.uptodate.com/contents/prescription-of-opioids-for-acute-pain-in-opioid-naive-patients#H1334178472

[13] Shah A, Hayes CJ, Martin BC. Factors influencing long-term opioid use among opioid naive patients: an examination of initial prescription characteristics and pain etiologies. J Pain. 2017;18(11):1374-1383. doi:10.1016/j.jpain.2017.06.010 28711636 PMC5660650

[14] Brat GA, Agniel D, Beam A, . Postsurgical prescriptions for opioid naive patients and association with overdose and misuse: retrospective cohort study. BMJ. 2018;360:j5790. doi:10.1136/bmj.j5790 29343479 PMC5769574

[15] The Society of Hospital Pharmacists of Australia. Reducing opioid-related harm: a hospital pharmacy landscape paper. Accessed May 16, 2019. https://www.shpa.org.au/sites/default/files/uploaded-content/website-content/shpa_-_reducing_opioid_related_harm.pdf

[16] Ballantyne JC, Kalso E, Stannard C. WHO analgesic ladder: a good concept gone astray. BMJ. 2016;352:i20. doi:10.1136/bmj.i20 26739664

[17] Janssen MF, Pickard AS, Golicki D, . Measurement properties of the EQ-5D-5L compared to the EQ-5D-3L across eight patient groups: a multi-country study. Qual Life Res. 2013;22(7):1717-1727. doi:10.1007/s11136-012-0322-4 23184421 PMC3764313

[18] US Centers for Disease Control and Prevention. Calculating total daily dose of opioids for safer dosage. Accessed September 7, 2018. https://www.cdc.gov/opioids/providers/prescribing/pdf/calculating-total-daily-dose.pdf

[19] Olsen MF, Bjerre E, Hansen MD, . Pain relief that matters to patients: systematic review of empirical studies assessing the minimum clinically important difference in acute pain. BMC Med. 2017;15(1):35. doi:10.1186/s12916-016-0775-3 28215182 PMC5317055

[20] Myles PS, Myles DB, Galagher W, . Measuring acute postoperative pain using the visual analog scale: the minimal clinically important difference and patient acceptable symptom state. Br J Anaesth. 2017;118(3):424-429. doi:10.1093/bja/aew466 28186223

[21] The Society of Hospital Pharmacists of Australia. Reducing opioid-related harm: a hospital pharmacy landscape paper. Accessed December 12, 2018. https://www.shpa.org.au/sites/default/files/uploaded-content/website-content/shpa_-_reducing_opioid_related_harm.pdf

[22] Chang AK, Bijur PE, Lupow JB, Gallagher EJ. Comparative analgesic efficacy of oxycodone/acetaminophen vs codeine/acetaminophen for short-term pain management following ED discharge. Pain Med. 2015;16(12):2397-2404. doi:10.1111/pme.12830 26176973

[23] Chang AK, Bijur PE, Munjal KG, John Gallagher E. Randomized clinical trial of hydrocodone/acetaminophen versus codeine/acetaminophen in the treatment of acute extremity pain after emergency department discharge. Acad Emerg Med. 2014;21(3):227-235. doi:10.1111/acem.12331 24628747

[24] Turturro MA, Paris PM, Yealy DM, Menegazzi JJ. Hydrocodone versus codeine in acute musculoskeletal pain. Ann Emerg Med. 1991;20(10):1100-1103. doi:10.1016/S0196-0644(05)81383-6 1928881

[25] Mitchell A, van Zanten SV, Inglis K, Porter G. A randomized controlled trial comparing acetaminophen plus ibuprofen versus acetaminophen plus codeine plus caffeine after outpatient general surgery. J Am Coll Surg. 2008;206(3):472-479. doi:10.1016/j.jamcollsurg.2007.09.006 18308218

[26] Mitchell A, McCrea P, Inglis K, Porter G. A randomized, controlled trial comparing acetaminophen plus ibuprofen versus acetaminophen plus codeine plus caffeine (Tylenol 3) after outpatient breast surgery. Ann Surg Oncol. 2012;19(12):3792-3800. doi:10.1245/s10434-012-2447-7 22713999

[27] Chang AK, Bijur PE, Esses D, Barnaby DP, Baer J. Effect of a single dose of oral opioid and nonopioid analgesics on acute extremity pain in the emergency department: a randomized clinical trial. JAMA. 2017;318(17):1661-1667. doi:10.1001/jama.2017.16190 29114833 PMC5818795

[28] Beaudoin FL. Combination of ibuprofen and acetaminophen is no different than low-dose opioid analgesic preparations in relieving short-term acute extremity pain. BMJ Evid Based Med. 2018;23(5):197-198. doi:10.1136/bmjebm-2018-110912 29700062

[29] Helmerhorst GTT, Zwiers R, Ring D, Kloen P. Pain relief after operative treatment of an extremity fracture: a noninferiority randomized controlled trial. J Bone Joint Surg Am. 2017;99(22):1908-1915. doi:10.2106/JBJS.17.00149 29135664

[30] Krebs EE, Gravely A, Nugent S, . Effect of opioid vs nonopioid medications on pain-related function in patients with chronic back pain or hip or knee osteoarthritis pain: the SPACE randomized clinical trial. JAMA. 2018;319(9):872-882. doi:10.1001/jama.2018.0899 29509867 PMC5885909

[31] Nüesch E, Rutjes AW, Husni E, Welch V, Jüni P. Oral or transdermal opioids for osteoarthritis of the knee or hip. Cochrane Database Syst Rev. 2009;(4):CD003115. doi:10.1002/14651858.CD003115.pub3 19821302

[32] Noble M, Treadwell JR, Tregear SJ, . Long-term opioid management for chronic noncancer pain. Cochrane Database Syst Rev. 2010;(1):CD006605. doi:10.1002/14651858.CD006605.pub2 20091598 PMC6494200

[33] Shaheed CA, Maher CG, McLachlan A. Investigating the efficacy and safety of over-the-counter codeine containing combination analgesics for pain and codeine based antitussives. Therapeutic Goods Administration. Accessed January 11, 2017. https://www.tga.gov.au/sites/default/files/review-efficacy-and-safety-over-counter-codeine-combination-medicines.pdf

