# Supplementary material for: Effectiveness of Oxycodone Hydrochloride (Strong Opioid) vs Combination Acetaminophen and Codeine (Mild Opioid) for Subacute Pain After Fractures Managed Surgically: A Randomized Clinical Trial
Source: JAMA Netw Open. 2021 Nov 17;4(11):e2134988. doi: 10.1001/jamanetworkopen.2021.34988 (PMC8600392; doi:10.1001/jamanetworkopen.2021.34988)
Supplement: Supplement 1. — Trial Protocol [file jamanetwopen-e2134988-s001.pdf]

Supplement 1. Ethics committee approved study protocol and study protocol manuscript

STUDY PROTOCOL

A Randomised Control Trial of Oxycodone Hydrochloride Versus Paracetamol with Codeine for Post-discharge Orthopaedic Fracture Pain

Principle Investigator: Deanne Jenkin
Supervising Investigator: Ian Harris
Supervising Investigator: Justine Naylor
Research Officer: Sarah Fares
Australian Clinical Trial Registration Number: ACTRN12616000941460

HREC approved protocol, version 3.0, Dated 27 July 2016

Background

Pain is an unavoidable part of the human experience. However, there is no way to objectively measure an individual's pain. It is a subjective experience, informed by each individual's particular physical, psychological, historical, social and cultural experiences and circumstances. Therefore, pain is often difficult to manage, and adequate pain relief is a major concern and area of focus in Australia today [1].

Postoperative pain is a major challenge in patients following orthopaedic surgery [2]. Serious risks for adverse events are evident with both opioid and nonsteroidal anti-inflammatory drugs (NSAIDs). Even though opioids are considered as the primary analgesic therapy in moderate to severe postoperative pain, they are associated with dose-related adverse effects such as sedation, respiratory depression, postoperative nausea and vomiting, pruritus, constipation and urinary retention [3, 4]. Furthermore the potential for misuse, abuse or addiction is also of concern [5, 6]. NSAIDs are associated with adverse effects such as gastrointestinal injury, increased operative site bleeding, renal toxicity, and bronchoconstriction [7, 8]. In addition, NSAIDs have been shown to interfere with fracture healing, bone-tendon healing, and spinal fusion [9, 10]. Paracetamol with its high safety profile in recommended dosage, lack of allergic potential and absence of contraindications in peptic ulcer diseases, haemostatic disorders, or pulmonary dysfunction has gained popularity as a complementary analgesic [11, 12]. Postoperative intravenous paracetamol has been found to reduce postoperative opioid consumption after orthopaedic surgery [2], but at present there is insufficient data to decide whether paracetamol reduces opioid-related adverse effects or if it provides equivalent pain management to stronger analgesics.

In orthopaedic osteoarthritis populations, level I evidence recommends not using opioids even if pain is severe [13]. The significant increase in the risk of adverse

events outweighs the small benefit of opioids which in turn limits their clinical importance and usefulness. In terms of post discharge analgesics there is a lack of evidence to inform practice. There are no randomised control trials directly comparing commonly prescribed analgesics and yet strong opioid prescription is common practice. Surprisingly, a recent randomised controlled study involving children with non-surgically managed fractures, showed that morphine (a strong opioid, taken orally) or ibuprofen improved pain scores with no significant difference in analgesic efficacy between the two approaches [14]. Morphine was associated with a greater frequency of adverse effects. If the same applies in adult populations, it is possible that we are overestimating the benefits of strong opioids and exposing patients to higher risk of adverse events. The current study will fill this gap in evidence and has the ability to change current practice or provide the evidence required to validate current practice.

Opioid analgesics are the drug of choice for post-operative pain fracture management [15]. From our previous research it has been found that most, if not all, surgically managed fracture patients are discharge home from hospital with strong opioids (largely Panadiene Forte, Endone and Oxycontin) [16]. Despite the overwhelming push for effective postoperative pain control, little evidence exists to inform optimal post-discharge pain management. There is no evidence for 'best choice' of opioid (mild or strong) or 'best' formulation (immediate- or controlled-release). Furthermore, evidence-based protocols, limits on dose, maximum prescription duration and patient education are deficient for this population. New, high quality evidence is required to help ensure patients' pain is adequately managed while costs and harms are minimised.

Methods/Design

This study is a double-blind, randomised controlled trial comparing Oxycodone hydrochloride Versus Paracetamol with Codeine for the Treatment of post discharge pain subsequent to orthopaedic fracture.

Participants and recruitment

Participants will be patients admitted to the orthopaedic ward who have sustained and require surgery for an orthopaedic fracture. Screening for participation will be undertaken by the research team who will obtain relevant medical history to assess the patient against the eligibility criteria. Eligible participants will be required to meet the following criteria: have sustained a non-pathological fracture of a long bone (humerus, radius, ulna, femur, tibia or fibula) or the pelvis, patella, calcaneus or talus treated with surgical fixation; be 18 years or older; and be able to comprehend the study protocol, written in English. Patients will be excluded if they meet any of the following criteria: have known or suspected multi-system trauma injuries (major head, chest or abdominal injury); have known or suspected major infection following surgery; have known or suspected opioid dependency; are pregnant or are breastfeeding; have contraindications to Oxycodone hydrochloride, Paracetamol or Codeine; or are taking an antidepressant or a sedative considered inappropriate in the opinion of investigators.

If a patient is considered eligible, a member of the research team will gain informed consent. The research team will provide education to the participant on how to take the study treatment, including providing the participant with a sealed medication

pack. A researcher (blinded) will collect baseline data from the participant prior to discharge. Following baseline data collection and prior to discharge from hospital the researcher will inform the participant to break the seal on the medication pack and commence the study medicine once discharged. At this point the participant is considered to be randomised into the study.

Randomisation and blinding

A researcher not involved in participant recruitment or data collection will generate a randomisation schedule a priori using a computer-derived random number sequence. Study medication will be over-encapsulated and prepared according to the randomisation schedule by the central study pharmacy, then sealed in medication blister packs and stored within the hospital pharmacy. Any medication over stock can be stored securely within a locked cabinet within the Principle Investigator office in the Research Institute until transfer to pharmacy occurs. All study medication will be accounted for and recorded on study pharmacy logs. The randomisation schedule will be kept concealed from other researchers and all participants. Following recruitment and upon discharge home from hospital, a hospital doctor will complete a study script which will be provided to pharmacy. Pharmacy will dispense a sealed medication pack (in chorological order) to the participant (blinded). The medication pack allocated to the participant can be collected by a nurse, doctor, delegated researcher or given directly to the participant prior to discharge, thus randomising the participant to one of two groups: oxycodone hydrochloride or paracetamol with codeine. The capsules for each group will be identical in appearance. The randomisation process will ensure concealed allocation and blinding of the researchers, participants and outcome assessors.

Study treatment

The dose of the study medication for the active group, Oxycodone hydrochloride immediate release, will be 5mg or 10mg four times daily or 1-2 tablets, providing a maximum dose of 40 mg daily or 8 tablets. The dose of the study medication for the control group, Paracetamol and Codeine phosphate, will be 500mg/8mg or 1000mg/14mg four times daily or 1-2 tablets, providing a maximum dose of 4000mg of Paracetamol and 64mg of codeine daily or 8 tablets.

In the standard study dosing regimen, the maximum tolerated dose for each participant will be maintained for 2 weeks before the study medication is titrated down to cessation in the final (3rd) treatment week. If adequate pain relief is achieved, defined as a pain rating of 0, 1 or 2 out of 10, for a minimum of 48 hours, before the 3-week standard treatment regimen is completed, early titration down to cessation is possible. The maximum treatment period is 3 weeks.

In addition to the study medicines, both groups may receive usual care during the 3-week treatment period. Usual care includes physical therapies. Other analgesic medications are prohibited during the treatment period unless approved by study doctors. If participants require more pain relief the participant will be discontinued from the study and referred to their general practitioner or orthopaedic surgeon for review of pain management going forward.

Data collection

Data collection will be conducted by the study researchers in person at baseline and via telephone at day 3 (3 days post discharge), and weeks 1, 2 and 3 post discharge. Data will be collected regardless of participants' compliance to the study treatment. Data collection by trained study researchers will increase data collection quality and aid participant retention.

Primary outcome

The primary outcome is average pain during week 1 of treatment measured using the Numerical Pain Rating Scale (NRS). The participants will be asked to rate their overall pain daily over the last 24 hours out of 10, with zero representing 'no pain', and 10 representing the 'worst pain imaginable'.

Secondary outcomes

The key secondary outcome is the EuroQol EQ-5D-5L measured at day 3 post discharge and weeks 1, 2 and 3 post discharge to assess quality of life. In addition, question 1 (mobility) and question 3 (usual activities) will be used to measure function. Pain will be measured daily using the Numerical Pain Rating Scale. The participants will be asked to rate their overall pain and worst pain over the last 24 hours out of 10, with zero representing 'no pain', and 10 representing the 'worst pain imaginable'. Medication adverse events will be measured daily using a list of common side effects experienced where participants answer yes or no. The Global Perceived Effect will be measured at weeks 1, 2, and 3, this asks the participant to compare their pain to when their injury first occurred. It is measured on a Likert scale, from a score of −5 (vastly worse) to 0 (unchanged), to +5 (completely recovered). Work and health utilisation questions will be asked at weeks 1, 2 and 3. These will report the use of health services and the return to paid employment following their injury.

Other data collected

Initial data (baseline) will be obtained directly from the patient after consent to the trial. This data will include age, gender, employment status, diagnoses, type of surgery, mechanism of injury, insurance status, pain and quality of life.

Details of adverse events will be collected at day 3 and weeks 1, 2 and 3 post-discharge. Any serious adverse events, defined as an event that is life threatening, results in death, hospitalisation, or significant disability, will be reported immediately to the Principle Investigator. The PI will investigate the relationship between a serious adverse event and the study medication, and if a potential relationship is suspected, unblinding to treatment allocation is permissible. The ethics committee will also be informed of the serious adverse event.

Questions will be asked regarding blinding and satisfaction at week 3. The blinding question will ask the participant to guess the treatment group to which they were randomised. The satisfaction question will ask the participant to rate how satisfied they felt with the study treatment overall on a 5-point scale (very dissatisfied, dissatisfied, neutral, satisfied or very satisfied).

Adherence to study medication will be ascertained via two methods: through participant documentation in a daily medication diary; and by counting the returned medicine, compared to the prescribed regimen. The latter is possible as participants

will be asked to return unused medicines via a reply-paid post satchel at the end of the 3-week treatment period.

Data integrity and analysis

The integrity of trial data will be monitored by regularly scrutinising data files for omissions and errors. Verification of source data will be performed by 100% data verification on all primary and secondary outcomes. The source of any inconsistencies will be explored and resolved. Electronic data will be stored on a secure server and paper copies located in a locked cabinet. Data will only be accessible by researchers and participant confidentiality will be maintained through secure data storage, during and post-trial.

The data will be analysed according to the intention-to-treat principle. Standard statistical techniques will be used to describe characteristics of patients in both groups. We will compare baseline characteristics in the two treatment groups and if incomparability appears, we will adjust for differences in secondary analysis. The primary outcome, average of pain over week 1, will be compared between groups using independent t-test. If adjustment for possible baseline incomparability is needed, analysis of covariance will be done. We will use the SAS statistical software package for data analysis. We consider p values less than 0.05 to be significant.

Sample size

The primary comparison is the difference between the two groups in average pain over the first 1 week, measured on an 11-point NPRS. A sample of 48 participants per group will provide 90% power to show at least a 1-unit (on a 11-point pain scale) advantage of Oxycodone hydrochloride over Paracetamol with Codeine based on a two-side type 2 error rate of 5%. Allowing for a loss to follow-up rate of 20%, a total of 116 participants will be required. A 1-point difference between the groups equates to an effect size of 0.67, assuming a standard deviation of 1.5 in the mean pain score in the control group.

Modification of the protocol

Any modifications to the protocol that may impact on the design and conduct of the study will require a formal protocol amendment. Such amendment will be agreed upon by the study investigators and approved by the ethics committee prior to implementation.

References

1. Nash R, Yates P, Edwards H, Fentiman B, Dewar A, McDowell J, Clark R. Pain and the administration of analgesia: what nurses say.Journal of Clinical Nursing. 8(2), 180-189.
2. Jebaraj B, Maitra S, Baidya DK, Puneet P. Intravenous Paracetamol Reduces Postoperative Opioid Consumption after Orthopedic Surgery: A Systematic Review of Clinical Trials. Pain Res Treat. 2013; 2013: 402510.
3. Weis OF, Sriwatanakul K, Alloza JL, Weintraub M, and Lasagna L. Attitudes of patients, housestaff, and nurses toward postoperative analgesic care. Anesthesia and Analgesia, vol. 62, no. 1, pp. 70–74, 1983.
4. Melzack R, Abbott FV, Zackon W, Mulder DS, Davis MW. Pain on a surgical ward: a survey of the duration and intensity of pain and the effectiveness of medication. Pain, vol. 29, no. 1, pp. 67–72, 1987.
5. Compton WM, Volkow ND. Major increases in opioid analgesic abuse in the United States: Concerns and strategies. Drug and Alcohol Dependence 81 (2006) 103–107.
6. Dobbin M. Pharmaceutical drug misuse in Australia. AustPrescr 2014;37:79-81
7. Kenny GN. Potential renal, haematological and allergic adverse effects associated with nonsteroidal anti-inflammatory drugs. Drugs, vol. 44, supplement 5, pp. 31–37, 1992.
8. Cashman J, McAnulty G. Nonsteroidal anti-inflammatory drugs in perisurgical pain management. Mechanisms of action and rationale for optimum use. Drugs, vol. 49, no. 1, pp. 51–70, 1995.
9. Glassman SD, Rose SM, Dimar JR, Puno RM, Campbell MJ, Johnson JR, The effect of postoperative nonsteroidal anti-inflammatory drug administration on spinal fusion. Spine, vol. 23, no. 7, pp. 834–838, 1998.
10. Lumawig JMT, Yamazaki A, Watanabe K. Dose-dependent inhibition of diclofenac sodium on posterior lumbar interbody fusion rates. Spine Journal, vol. 9, no. 5, pp. 343–349, 2009.
11. Ivey KJ. Gastrointestinal effects of antipyretic analgesics. The American Journal of Medicine, vol. 75, no. 5 A, pp. 53–64, 1983.
12. Mielke CH. Comparative effects of aspirin and acetaminophen on hemostasis. Archives of Internal Medicine, vol. 141, no. 3, pp. 305–310, 1981.
13. da Costa BR, Nüesch E, Kasteler R, Husni E, Welch V, Rutjes AWS, Jüni P. Opioids for osteoarthritis. Cochrane library. Published 17 September 2014
14. Poonai N et al.Oral administration of morphine versus ibuprofen to manage postfracture pain in children: a randomized trial. *CMAJ December 9, 2014 vol. 186 no.18*First doi: 10.1503/cmaj.140907
15. Roberts LJ. Managing acute pain in patients with an opioid abuse or dependence disorder. AustPrescr 2008;31:133-5.
16. Jenkin DE, Naylor JM, Harris IA. Opioid dependence after major fracture. Under review

Oxycodone Hydrochloride (Strong Opioids) versus Combination Paracetamol plus Codeine (Mild Opioids) for Post-discharge Orthopaedic Fracture Pain: A Randomised Controlled Trial Protocol

DE Jenkin, BSc, MClinT(R)[1,2,3]

JM Naylor, BAppSc (Phty), PhD[1,2,3,4]

J Descallar. BSc, MBiostat[1,3]

IA Harris, MBBS, MMed(ClinEpi), PhD, FRACS(Orth), FAOrthA, FAHMS[1,2,3,4]

1. UNSW Australia
2. Whitlam Orthopaedic Research Centre
3. Ingham Institute for Applied Medical Research
4. Liverpool Hospital, South Western Sydney Local Health District, NSW, Australia

Protocol manuscript approved by authors: 27 March 2017

ABSTRACT

***Background*:** Opiates have been used for centuries and remain the most potent and reliable analgesic agents for acute pain. Orthopaedic surgeons are among the top prescribers of opioid analgesics as severe postoperative pain is common following orthopaedic surgery. Surgically managed fracture patients are commonly discharged home from hospital with a strong opiate prescription, yet little evidence exists to guide this approach. While adverse events and the risk of mortality rise proportionally with the opioid dose, analgesic and functional benefits do not. Thus, discharge home from hospital with strong opiate prescription should be considered carefully. We propose that discharge from hospital signifies the most suitable time point to transition people down the World Health Organisation analgesic ladder from step 3 (strong opioid) to step 2 (mild opioid). The primary goal of conducting this randomised control trial is to test the superiority of strong opioids (5mg oxycodone) over mild opioids (8mg codeine with 30 mg paracetamol) for management of pain after discharge from acute care in an orthopaedic trauma cohort.

Design: A double-blind randomised controlled trial of paracetamol plus codeine (Mild opioids) versus oxycodone (Strong opioids) for the treatment of pain after discharge from orthopaedic surgical fracture management.

Methods: One hundred and twenty (120) participants will be randomly assigned to one of two treatment arms: mild opioids (8mg codeine with 30mg paracetamol) or strong opioids (5mg short acting oxycodone) at discharge from hospital. Participants will be evaluated over a 3-week program or until recovery. Participants will be asked to fill in a daily medication and pain diary and will be contacted at days 3, 7, 14 and 21 post-discharge for assessment of outcomes (pain, daily analgesic usage, adverse events, quality of life, return to work and perceived effect)

Discussion: The results of the RCT will provide information about the comparative effectiveness and harms of strong opioids versus mild opioids and evaluate the suitability of dose reduction at discharge after acute care.

KEYWORDS

Fracture; Orthopaedic Trauma; Pain; Analgesics; Opioid; Narcotics.

TRIAL REGISTRATION

Australian and New Zealand Clinical Trial Registration, Number: ACTRN12616000941460

BACKGROUND

Opiates have been used for centuries and remain to this day the most potent and reliable analgesic agents.[1.] Evidence supports the use of opioids to relieve moderate to severe pain, particularly acute and cancer pain.[2]. In these time-limited situations, the efficacy of opiates is extensively documented and broadly accepted. Evidence does not support using opioid therapy for chronic pain. However, the prescribing of opioids for chronic pain is increasing and evidence is growing of the adverse effects of their long-term use.[4-6] Their use has recently grown, in part because providing adequate pain relief is now considered an important standard of care.[7] While there is little debate over the short-term use of opiates, their use for chronic non-malignant pain is controversial and there is growing reluctance among some physicians to prescribe them. The problem is that the most powerful opiate analgesics are also the most liable to cause abuse and addiction.[3]

Orthopaedic surgeons are among the top prescribers of opioid analgestics.[9] Severe postoperative pain is common following orthopaedic surgery, and its safe and effective management can be challenging for everyone on the health care team.[10] Currently, an array of choices are available to orthopaedic surgeons for acute postoperative pain management,[11] but oral analgesics are usually prescribed for ongoing pain management post discharge.

Although opioids are considered as the primary analgesic therapy in moderate to severe postoperative pain, they are associated with dose-related adverse effects such as sedation, respiratory depression, postoperative nausea and vomiting, pruritus, constipation and urinary retention.[12, 13] Furthermore the potential for misuse, abuse or addiction is also of concern.[14, 15] NSAIDs are commonly used post-operatively but are associated with adverse effects such as gastrointestinal injury, increased operative site bleeding, renal toxicity, and bronchoconstriction.[16, 17] In addition, NSAIDs have been shown to interfere with fracture healing, bone-tendon healing, and spinal fusion.[18-20] Paracetamol with its high safety profile in recommended dosage, lack of allergic potential and absence of contraindications in peptic ulcer diseases, haemostatic disorders, or pulmonary dysfunction has gained popularity as a

complementary analgesic.[21, 22] Postoperative intravenous paracetamol has been found to reduce postoperative opioid consumption after orthopaedic surgery,[22] but at present there is insufficient data to decide whether paracetamol reduces opioid-related adverse effects or if it provides equivalent pain management to stronger analgesics counterparts. Codeine is a 'weak' opioid, classed as step two of the World Health Organisation (WHO) analgesic ladder. Results from individual trials suggest that codeine and paracetamol combination medicines provide greater pain relief versus placebo and that these results are generally superior to the pain relief effects obtained from combination of codeine and NSAIDs versus placebo.[23] Codeine also presents a lower risk from opioid related death as for every two Schedule 8 (**Schedule II** narcotic**s** in US) opioid-related deaths in 2009, there was only one codeine-related death in Australia. [24]

Opioid analgesics are the drug of choice for post-operative pain fracture management[26-28] and in Australia anecdotal evidence suggests most surgically managed fracture patients are discharged home from hospital with a strong opiate prescription. Despite the demand for effective postoperative pain control, little evidence exists for post-discharge pain management. There is no evidence for 'best choice' of opioid (mild or strong) or 'best' formulation (immediate- or controlled- release). Furthermore, evidence-based protocols, limits on dose, maximum prescription duration and patient education are deficient for this population. In considering that 13% of people receiving a strong opioid took the opioid for six or more weeks, strong opioid prescription following discharge from hospital should be considered carefully.[24] In addition, while adverse events and the risk of mortality rise proportionally with the opioid dose, analgesic and functional benefits may not[29,30]. The mortality risk rises without increased benefit from a daily opioid dose of more than 100mg of oral morphine or the equivalent.[31] We propose that discharge from hospital signifies the most suitable time point to transition people down the WHO analgesic ladder from step 3 (strong opioid) to step 2 (mild opioid) analgesics. We hypothesise that a strong opioid will provide better analgesia than a mild opioid–paracetamol combination when given post-discharge in orthopaedic surgical patients.

METHODS

Design

This study is a double-blind, randomised controlled trial comparing Oxycodone hydrochloride (5mg) versus Paracetamol with Codeine (500mg/8mg) for the Treatment of post discharge pain subsequent to orthopaedic fracture.

Participants and recruitment

Participants will be patients admitted to the orthopaedic ward with an acute fracture requiring surgery. Screening for participation will be undertaken by the research team who will obtain relevant medical history to assess the patient against the eligibility criteria. Eligible participants will be required to meet the following criteria: have sustained a non-pathological fracture of a long bone (humerus, radius, ulna, femur, tibia or fibula) or the pelvis, patella, calcaneus or talus, treated with surgical fixation; be 18 years of age or older; and be able to comprehend the study protocol, written in English. Patients will be excluded if they meet any of the following criteria: have known or suspected multi-system trauma injuries (major head, chest or abdominal injury); have known or suspected major infection following surgery; have known or suspected opioid dependency; are pregnant or are breastfeeding; have contraindications to oxycodone hydrochloride, paracetamol or codeine; or are taking an antidepressant or a sedative.

If a patient is considered eligible, a member of the research team will gain informed consent. The research team will provide education to the participant on how to take the study treatment, including providing the participant with a sealed medication pack. A researcher (blinded) will collect baseline data from the participant prior to discharge. Following baseline data collection and prior to discharge from hospital the researcher will inform the participant to break the seal on the medication pack and commence the study medicine once discharged. At this point the participant is considered to be randomised on the study.

Randomisation and blinding

A researcher not involved in participant recruitment or data collection will generate a randomisation schedule a priori using a computer-derived random number sequence. Study medication in both groups will be identically over-encapsulated and prepared according to the randomisation schedule by the central study pharmacy, then sealed in medication blister packs and stored within the hospital pharmacy. All study medication will be accounted for and recorded on study pharmacy logs. The randomisation schedule will be kept concealed from other researchers and all participants. Following recruitment and upon discharge home from hospital, a hospital doctor will complete a study script which will be provided to pharmacy. Pharmacy will dispense a sealed medication pack (numerical order) to the participant. Upon opening of the medication pack following discharge from hospital the participant is randomised to one of two groups: oxycodone hydrochloride or paracetamol with codeine. The randomisation process will ensure concealed allocation. Researchers, participants and outcome assessors will be blinded. Statistical analysis and reporting and preparation of the results and discussion (two versions) will be performed prior to unblinding.

Study treatment

The dose of the study medication for the active group, oxycodone hydrochloride immediate release, will be 5mg or 10mg (1 – 2 tablets) four times per day, providing a maximum dose of 40 mg daily or 8 tablets. The dose of the study medication for the control group, paracetamol and codeine phosphate, will be 500mg/8mg or 1000mg/14mg (1 – 2 tablets) four times per day, providing a maximum dose of 4000mg of paracetamol and 64mg of codeine daily or 8 tablets.

In the standard study dosing regimen, the maximum tolerated dose for each participant will be maintained for 2 weeks before the study medication is titrated down to cessation in the final (3rd) treatment week. If adequate pain relief is achieved, defined as a pain rating of 0, 1 or 2 out of 10, for a minimum of 48 hours or if the participant considers pain well controlled before the 3-week standard treatment regimen is completed, early titration down to cessation is allowed. The maximum treatment period is 3 weeks.

In addition to the study medicines, both groups will receive usual care during the 3-week treatment period. Usual care includes physical therapies. Other analgesic medications are prohibited during the treatment period except NSAIDs approved by study doctors. If participants require more pain relief the participant will be discontinued from the study and referred to their general practitioner or orthopaedic surgeon for review of pain management.

Data collection

Data collection will be conducted by the study researchers in person at baseline and via telephone at day 3 (3 days post discharge), and weeks 1, 2 and 3 post discharge. Baseline data will be collected on the hospital ward prior to discharge. Data will be collected regardless of participants' compliance to the study treatment. Data collection by trained study researchers will increase data collection quality and aid participant retention.

Primary outcome

The primary outcome is average daily pain during week 1 of treatment measured using the Numerical Pain Rating Scale (NRS). The participants will be asked to rate their average pain daily over the last 24 hours out of 10, with zero representing 'no pain', and 10 representing the 'worst pain imaginable'.

Secondary outcomes

The key secondary outcome is the EuroQol EQ-5D-5L measured at day 3 post discharge and weeks 1, 2 and 3 post discharge to assess quality of life. In addition, question 1 (mobility) and question 3 (usual activities) will be used to measure function. Worst pain will be measured daily using the Numerical Pain Rating Scale. The participants will be asked to rate their worst pain over the last 24 hours out of 10, with zero representing 'no pain', and 10 representing the 'worst pain imaginable'. Note that this outcome (worst pain over the last 24 hours) should be distinguished from the primary outcome of average pain over the last 24 hours. Medication adverse events will be measured daily (and recorded in the pain diary) using a list of common side effects experienced where participants

answer yes or no. The Global Perceived Effect will be measured at weeks 1, 2, and 3, this asks the participant to compare their pain to when their injury first occurred. It is measured on a Likert scale, from a score of −5 (vastly worse) to 0 (unchanged), to +5 (completely recovered). Return to work (Yes [full or light duties] or No) will be asked at weeks 1, 2 and 3. These will report the return to paid employment rate, capacity and time point following their injury.

Other data collected

Descriptive data including age, gender, employment status, diagnoses, type of surgery, mechanism of injury, insurance status, pain and quality of life, will also be collected on entry to study.

Details of adverse events will be collected at day 3 and weeks 1, 2 and 3 post-discharge. Any serious adverse events, defined as an event that is life threatening, results in death, hospitalisation, or significant disability, will be reported immediately to the Principal Investigator (PI). The PI will investigate the relationship between a serious adverse event and the study medication, and if a potential relationship is suspected, unblinding to treatment allocation is permissible. The ethics committee will also be informed of the serious adverse event.

Questions will be asked regarding blinding and satisfaction at week 3. The blinding question will ask the participant to guess the treatment group to which they were randomised. The satisfaction question will ask the participant to rate how satisfied they felt with the study pain management on a 5-point scale (very dissatisfied, dissatisfied, neutral, satisfied or very satisfied).

Adherence to study medication will be ascertained via two methods: through participant documentation in a daily medication diary; and by counting the returned medicine, compared to the prescribed regimen. The latter is possible as participants will be asked to return unused medicines via a reply-paid post satchel at the end of the 3-week treatment period.

Data integrity and analysis

The integrity of trial data will be monitored by regularly scrutinising data files for omissions and errors. Verification of source data will be performed by 100% data verification on all primary and secondary outcomes. The source of any inconsistencies will be explored and resolved. Electronic data will be stored on a secure server and paper copies located in a locked cabinet. Data will only be accessible by researchers and participant confidentiality will be maintained through secure data storage, during and post-trial.

The distribution of characteristics of patients included in the study will be presented in a table comparing baseline characteristics between the treatment arms. Significance testing to compare baseline covariates between treatment arms will not be utilised. Data will be analysed by a statistician who is blinded to group status. All analyses will be by intention-to-treat and missing data will not be substituted unless there is more than 5% missing data for any variable in which case we will use Multiple Imputation. The primary analysis will be a multilevel model comparing daily pain scores between treatment groups whilst accounting for repeated measures by patient. Secondary analyses will include a pre-protocol analysis of the primary outcome and unadjusted analysis of the secondary outcomes using multilevel models. For primary outcome, a p value of <0.05 will be considered statistically significant.

Sample size

The primary comparison is the difference between the two groups in average pain over the first 1 week, measured on an 11-point NRS. A sample of 46 participants per group (322 individual NRS pain scores) will provide greater than 90% power to show at least a 1.0-unit (on a 10-point pain scale) advantage of oxycodone over paracetamol plus codeine assuming an overall SD of 3 for the daily NRS score, an ICC of 0.25 (ie. we expect 25% of the variation in NRS score to be between patients) and a two-sided type 1 error rate of 5%. A sample of 60 participants per group (120 total) will be collected to allow for a loss to follow-up rate of 20%.

DISCUSSION

Despite the demand for effective postoperative pain control, little evidence exists for post-discharge pain management. There is no evidence for 'best choice' of opioid (mild or strong) or 'best' formulation (immediate- or controlled- release). Furthermore, evidence-based protocols, limits on dose, maximum prescription duration and patient education are deficient for this population. The results of the RCT will provide information about the superiority of strong opioids over mild opioids and evaluate the suitability of dose reduction at discharge after acute care.

ETHICAL APPROVAL

Hunter New England Health Research Ethics Committee, NSW, Australia, approved 14th July 2015, Reference: HREC 15/05/20/3.02

AVAILABILITY OF DATA

The datasets used and/or analysed during the current study are available from the corresponding author on reasonable request.

FUNDING

This trial has received competitive grant funding from the New South Wales Government. Australia, State Insurance Regulatory Authority (formally known as the NSW Motor Accident Authority) Acute Trauma Grant Round, Ref: 15/250.

The funder has no role in the design of the study and collection, analysis, and interpretation of data and in writing of the manuscripts.

AUTHORS' CONTRIBUTIONS

DJ, JN and IH were major contributors to trial and protocol design, grant writing and funding. DJ, JN JD and IH wrote the protocol manuscript. All authors read and approved the final manuscript on 27 March 2017.

REFERENCES

1. Pasternak GW. The Opiate Receptors. Humana Press/Springer; New York: 2011.
2. Macintyre P, Schug S, Scott D, Visser E, Walker S. Acute Pain Management: Scientific Evidence (3rd edition). APM: SE Working Group of the Australian and New Zealand College of Anaesthetists and Faculty of Pain Medicine. Melbourne: ANZCA & FPM, 2010.
3. Fields HL. The Doctor's Dilemma: opiate analgesics and chronic pain. *Neuron*. 2011;69(4):591-594. doi:10.1016/j.neuron.2011.02.001.
4. Hunter Integrated Pain Service. Reconsidering Opioid Therapy. A Hunter New England Perspective. Newcastle: HIPS, 2014.
5. Nicholas R, Lee N, Roche A. Pharmaceutical Drug Misuse in Australia: Complex Problems, Balanced Responses. Adelaide: National Centre for Education and Training on Addiction (NCETA), Flinders University, 2011.
6. Blanch B, Pearson SA, Haber PS. An overview of the patterns of prescription opioid use, costs and related harms in Australia. BJCP. 2014;78(5):1159–66.
7. Wells N, Pasero C, McCaffery M. Improving the Quality of Care Through Pain Assessment and Management. In: Hughes RG, editor. Patient Safety and Quality: An Evidence-Based Handbook for Nurses. Rockville (MD): Agency for Healthcare Research and Quality (US); 2008 Apr. Chapter 17. Available from: https://www.ncbi.nlm.nih.gov/books/NBK2658/
8. Blake H, Leighton P, van der Walt G, Ravenscroft A. Prescribing opioid analgesics for chronic non-malignant pain in general practice – a survey of attitudes and practice. *British Journal of Pain*. 2015;9(4):225-232. doi:10.1177/2049463715579284.
9. Volkow ND, McLellan TA, Cotto JH, Karithanom M, Weiss SR: Characteristics of opioid prescriptions in 2009. JAMA 2011;305(13):1299–1301.
10. Pasero C, McCaffery M. Orthopaedic postoperative pain management**.** J Perianesth Nurs. 2007 Jun;22(3):160-72; quiz 172-3.
11. Herkowitz H, Dirschl D, Sohn D. Pain Management: The Orthopaedic Surgeon's Perspective, J Bone Joint Surg Am. 2007;89:2532-5

12. Weis OF, Sriwatanakul K, Alloza JL, Weintraub M, and Lasagna L. Attitudes of patients, housestaff, and nurses toward postoperative analgesic care. Anesthesia and Analgesia, vol. 62, no. 1, pp. 70–74, 1983.
13. Melzack R, Abbott FV, Zackon W, Mulder DS, Davis MW. Pain on a surgical ward: a survey of the duration and intensity of pain and the effectiveness of medication. Pain, vol. 29, no. 1, pp. 67–72, 1987.
14. Compton WM, Volkow ND. Major increases in opioid analgesic abuse in the United States: Concerns and strategies. Drug and Alcohol Dependence 81 (2006) 103–107.
15. Dobbin M. Pharmaceutical drug misuse in Australia. AustPrescr 2014;37:79-81
16. Kenny GN. Potential renal, haematological and allergic adverse effects associated with nonsteroidal anti-inflammatory drugs. Drugs, vol. 44, supplement 5, pp. 31–37, 1992.
17. Cashman J, McAnulty G. Nonsteroidal anti-inflammatory drugs in perisurgical pain management. Mechanisms of action and rationale for optimum use. Drugs, vol. 49, no. 1, pp. 51–70, 1995.
18. Glassman SD, Rose SM, Dimar JR, Puno RM, Campbell MJ, Johnson JR, The effect of postoperative nonsteroidal anti-inflammatory drug administration on spinal fusion. Spine, vol. 23, no. 7, pp. 834–838, 1998.
19. Lumawig JMT, Yamazaki A, Watanabe K. Dose-dependent inhibition of diclofenac sodium on posterior lumbar interbody fusion rates. Spine Journal, vol. 9, no. 5, pp. 343–349, 2009.
20. Ivey KJ. Gastrointestinal effects of antipyretic analgesics. The American Journal of Medicine, vol. 75, no. 5 A, pp. 53–64, 1983.
21. Mielke CH. Comparative effects of aspirin and acetaminophen on hemostasis. Archives of Internal Medicine, vol. 141, no. 3, pp. 305–310, 1981
22. Jebaraj B, Maitra S, Baidya DK, Puneet P. Intravenous Paracetamol Reduces Postoperative Opioid Consumption after Orthopedic Surgery: A Systematic Review of Clinical Trials. Pain Res Treat. 2013; 2013: 402510.

23. Shaheed C, Maher C & McLachlan A. Investigating the efficacy and safety of over-the-counter codeine containing combination analgesics for pain and codeine based antitussives. Australia: Therapeutic Goods Administration; 2016. Available at: https://www.tga.gov.au/sites/default/files/review-efficacy-and-safety-over-counter-codeine-combination-medicines.pdf (accessed 11 January 2017)
24. *The Australian Commission on Safety and Quality in Health Care. Australian Atlas of Healthcare Variation. 201, chapter 5. available at: https://www.safetyandquality.gov.au/atlas/ (assessed 16 March 2017)*
25. Roxburgh A, Hall WD, Burns L, Pilgrim J, Saar E, Nielsen S and Degenhardt L. Trends and characteristics of accidental and intentional codeine overdose deaths in Australia Med J Aust 2015; 203 (7): 299.
26. Helmerhorst GT, Lindenhovius AL, Vrahas M, Ring D, Kloen P. Satisfaction with pain relief after operative treatment of an ankle fracture. Injury. 2012;43:1958–1961. doi: 10.1016/j.injury.2012.08.018.
27. Trevino CM, deRoon-Cassini T, Brasel K. Does opiate use in traumatically injured individuals worsen pain and psychological outcomes? *J Pain.* 2013;14:424–430.
28. Garimella V, Cellini C. Postoperative Pain Control. *Clinics in Colon and Rectal Surgery*. 2013;26(3):191-196. doi:10.1055/s-0033-1351138.
29. Bohnert AS, Valenstein M, Bair MJ, Ganoczy D, McCarthy JF, Ilgen MA et al. Association between opioid prescribing patterns and opioid overdose-related deaths. JAMA 2011;305(13):1315–21.
30. Chen L, Vo T, Seefeld L, Malarick C, Houghton M, Ahmed S, Zhang Y, Cohen A, Retamozo C, St Hilaire K, Zhang V, Mao J. Lack of correlation between opioid dose adjustment and pain score change in a group of chronic pain patients. J Pain. 2013;14:384–392. doi: 10.1016/j.jpain.2012.12.012.
31. NSW Agency for Clinical Innovation (ACI) Pain Management Network. Opioid recommendations in General Practice. Sydney: ACI, 2014
