# Supplementary material for: Effectiveness of Oxycodone Hydrochloride (Strong Opioid) vs Combination Acetaminophen and Codeine (Mild Opioid) for Subacute Pain After Fractures Managed Surgically: A Randomized Clinical Trial
Source: JAMA Netw Open. 2021 Nov 17;4(11):e2134988. doi: 10.1001/jamanetworkopen.2021.34988 (PMC8600392; doi:10.1001/jamanetworkopen.2021.34988)
Supplement: Supplement 2. — eAppendix. Extract From Participant Daily Diary eTable 1. Baseline Characteristics for Participants Giving Consent: Randomized vs Nonrandomized eTable 2. Reported Daily Adverse Effects eTable 3. Quality of Life Scores for EuroQol 5-Dimension 5-Level Questionnaire Dimensions at Days 3 and 7 [file jamanetwopen-e2134988-s002.pdf]

Supplemental Online Content

Jenkin DE, Naylor JM, Descallar J, Harris IA. Effectiveness of oxycodone hydrochloride (strong opioid) vs combination acetaminophen and codeine (mild opioid) for subacute pain after fractures managed surgically: a randomized clinical trial. *JAMA Netw Open*. 2021;4(11):e2134988. doi:10.1001/jamanetworkopen.2021.34988

eAppendix. Extract From Participant Daily Diary

eTable 1. Baseline Characteristics for Participants Giving Consent: Randomized vs Nonrandomized

eTable 2. Reported Daily Adverse Effects

eTable 3. Quality of Life Scores for EuroQol 5-Dimension 5-Level Questionnaire Dimensions at Days 3 and 7

This supplemental material has been provided by the authors to give readers additional information about their work.

© 2021 Jenkin DE et al. *JAMA Network Open.*

eAppendix. Extract From Participant Daily Diary

Pain diary
Recording your pain

Maintaining information about your pain and how it affects your lifestyle will help you and your health professionals understand any patterns. This will help them work with you to manage your pain better.

Please complete the following table for each day, for 21 days in a row. The record will also help monitor the progress of treatment and any patterns of improvement.

To fill in the table, it is important to write a score between 0–10 for average pain and worst pain once per day in the afternoon to get a better understanding about how your pain medicine effectiveness and frequency affects your pain.

For example, to show how your average pain in the last 24 hours is, it is important to rate your pain. A zero (0) means no pain, and ten (10) means worst possible pain.

Select one number that best describes your pain:

0	1	2	3	4	5	6	7	8	9	10
No pain										Worst possible pain

© 2021 Jenkin DE et al. *JAMA Network Open.*

Day 1: Date: ____________________

Average pain	Pain 0-10 (*please circle one number below)*
**Afternoon**<br>**Time:__ __ : __ __ PM**	0 1 2 3 4 5 6 7 8 9 10

Worst pain	Pain 0-10 (*please circle one number below)*
**Afternoon**<br>**Time:__ __ : __ __ PM**	0 1 2 3 4 5 6 7 8 9 10

Have you experience any side effects today? *(Please circle answer)* Yes No

If ***yes*** please circle all experienced symptoms from the list below:

Constipation Drowsiness Nausea Vomiting Hallucinations Dizziness

Confusion Skin rash Other: ____

Day 2: Date: ____________________

Average pain	Pain 0-10 (*please circle one number below)*
**Afternoon**<br>**Time:__ __ : __ __ PM**	0 1 2 3 4 5 6 7 8 9 10

Worst pain	Pain 0-10 (*please circle one number below)*
**Afternoon**<br>**Time:__ __ : __ __ PM**	0 1 2 3 4 5 6 7 8 9 10

Have you experience any side effects today? *(Please circle answer)* Yes No

If ***yes*** please circle all experienced symptoms from the list below:

Constipation Drowsiness Nausea Vomiting Hallucinations Dizziness

Confusion Skin rash Other: ____

© 2021 Jenkin DE et al. *JAMA Network Open.*

eTable 1. Baseline Characteristics for Participants Giving Consent: Randomized vs Nonrandomized

Characteristic	Consented and randomized n=120	Consented but not randomized n=14
Men, No. (%)	90 (75)	12 (86)
Age, mean (SD), y	37 (14)	37 (15)
Total Fractures, No. (%)		
1	105 (88)	13 (93)
≥ 2	15 (12)	1 (7)
Mechanism of Injury, No. (%)		
Road related trauma	35 (29)	3 (21)
Fall	42 (35)	4 (29)
Blunt or crush trauma	41 (34)	6 (43)
Other	2 (2)	0 (0)
Region of Fracture, No. (%)		
Upper Extremity[a]	34 (28)	5 (36)
Lower Extremity[b]	67 (56)	7 (50)
Pelvis	4 (3)	1 (7)
Multiple fractures regions	15 (13)	1 (7)

[a] includes: humerus, radius/ulna. [b]includes: femur, patella, tibia/fibula, calcaneus/talus.

© 2021 Jenkin DE et al. *JAMA Network Open.*

eTable 2. Reported Daily Adverse Effects

	Number of participants reporting ≥1 daily side effect[b]	
**Study day[a]**	**Oxycodone[c]**	**Acetaminophen & codeine[d]**
day, n (%)		
1	25 (42.4)	33 (54.1)
2	32 (54.2)	34 (55.7)
3	32 (54.2)	27 (44.3)
4	33 (55.9)	21 (34.4)
5	31 (52.5)	19 (31.1)
6	30 (50.8)	16 (26.2)
7	28 (47.5)	17 (27.8)
8	11 (23.9)	5 (10.9)
9	8 (17.4)	5 (10.9)
10	6 (13.0)	5 (10.9)
11	7 (15.2)	6 (13.0)
12	11 (23.9)	8 (17.4)
13	9 (20.0)	7 (15.2)
14	12 (26.1)	5 (10.8)
15	6 (28.6)	2 (6.9)
16	7 (33.3)	4 (13.8)
17	4 (19.0)	5 (17.2)
18	4 (19.0)	4 (13.8)
19	7 (33.3)	2 (6.9)
20	5 (23.8)	3 (10.3)
21	5 (23.8)	1 (3.4)

[a]Primary outcome period includes days 1 to 7 (week 1); secondary outcome period includes days 8 to 14 (week 2) and days 15 to 21 (week 3). [b]Participants reported any experienced study medication side effect daily using a diary.[c]The oxycodone group (strong opioid) had 59, 46, 21 participants start week 1, 2 and 3, respectively. [d]The acetaminophen & codeine group (mild opioid) had 61, 46, 29 participants start week 1, 2 and 3, respectively.

© 2021 Jenkin DE et al. *JAMA Network Open.*

eTable 3. Quality of Life Scores for EuroQol 5-Dimension 5-Level Questionnaire Dimensions at Days 3 and 7

Outcome	Total no. of participants		P-value[a]
	Oxycodone	Acetaminophen & Codeine	
**EQ-5D, Day 3, No (%)**			
**Mobility**			
No problems	22 (37.9)	22 (36.1)	
Slight problems	9 (15.5)	10 (16.4)	
Moderate problems	23 (39.7)	17 (27.9)	
Severe problems	4 (6.9)	10 (16.4)	
Unable	0 (0.0)	2 (3.3)	0.45
**Self care**			
No problems	9 (15.5)	9 (14.8)	
Slight problems	19 (32.8)	17 (27.9)	
Moderate problems	19 (32.8)	23 (37.7)	
Severe problems	11 (19.0)	11 (18.0)	
Unable	0 (0.0)	1 (1.6)	0.21
**Usual Activities**			
No problems	1 (1.7)	1 (1.6)	
Slight problems	6(10.3)	8 (13.1)	
Moderate problems	15 (25.9)	12 (19.7)	
Severe problems	18 (31.0)	13 (21.3)	
Unable	18 (31.0)	27 (44.3)	0.62
**Pain/discomfort**			
No pain or discomfort	0 (0.0)	1 (1.6)	
Slight pain or discomfort	15 (25.9)	14 (23.0)	
Moderate pain or discomfort	38 (65.5)	32 (52.5)	
Severe pain or discomfort	5 (8.6)	13 (21.3)	
Extreme pain or discomfort	0 (0.0)	1 (1.6)	0.36
**Anxiety/depression**			
Not anxious or depressed	42 (72.4)	38 (62.3)	
Slightly anxious or depressed	12 (20.7)	14 (23)	
Moderately anxious or depressed	2 (3.45)	7 (11.5)	
Severely anxious or depressed	2 (3.45)	2 (3.3)	
Extremely anxious or depressed	0 (0.0)	0 (0.0)	0.19
**EQ-5D, Day 7, No (%)**			
**Mobility**			
No problems	29 (49.2)	19 (31.7)	
Slight problems	8 (13.6)	21 (35.0)	
Moderate problems	18 (30.5)	14 (23.3)	
Severe problems	4 (6.8)	4 (6.7)	
Unable	0 (0.0)	2 (3.3)	0.29
**Self care**			
No problems	9 (15.2)	13 (21.7)	
Slight problems	26 (44.1)	22 (36.7)	
Moderate problems	19 (32.2)	21 (35.0)	
Severe problems	3 (5.1)	3 (5.0)	
Unable	2 (3.4)	1 (1.7)	0.28
**Usual Activities**			
No problems	1 (1.7)	1 (1.7)	
Slight problems	7 (11.9)	12 (20.0)	
Moderate problems	24 (40.7)	18 (30.0)	
Severe problems	17 (28.8)	12 (20.0)	
Unable	10 (17)	17 (28.3)	0.69
**Pain/discomfort**			
No pain or discomfort	4 (6.8)	2 (3.3)	
Slight pain or discomfort	25 (42.4)	24 (40.0)	
Moderate pain or discomfort	28 (47.4)	28 (46.7)	
Severe pain or discomfort	2 (3.4)	6 (10.0)	
Extreme pain or discomfort	0 (0.0)	0 (0.0)	0.78
**Anxiety/depression**			
Not anxious or depressed	45 (76.3)	37 (61.7)	
Slightly anxious or depressed	10 (17)	16 (26.7)	
Moderately anxious or depressed	3 (5.1)	6 (10.0)	
Severely anxious or depressed	0 (0.0)	1 (1.7)	
Extremely anxious or depressed	1 (1.7)	0 (0.0)	0.09

[a]analyzed by generalized estimating equation with cumulative logit link.

© 2021 Jenkin DE et al. *JAMA Network Open.*
