# Supplementary material for: Effectiveness of Oxycodone Hydrochloride (Strong Opioid) vs Combination Acetaminophen and Codeine (Mild Opioid) for Subacute Pain After Fractures Managed Surgically: A Randomized Clinical Trial
Source: JAMA Netw Open. 2021 Nov 17;4(11):e2134988. doi: 10.1001/jamanetworkopen.2021.34988 (PMC8600392; doi:10.1001/jamanetworkopen.2021.34988)
Supplement: Supplement 3. — Data Sharing Statement [file jamanetwopen-e2134988-s003.pdf]

Data Sharing Statement

Jenkin DE, Naylor JM, Descallar J, Harris IA. Effectiveness of oxycodone hydrochloride (strong opioid) vs combination acetaminophen and codeine (mild opioid) for subacute pain after fractures managed surgically: a randomized clinical trial. JAMA Netw Open. 2021;4(11):e2134988. doi:10.1001/jamanetworkopen.2021.34988

Data

Data available: No

Additional Information

Explanation for why data not available: The datasets used and/or analyzed during this study are available from the corresponding author upon reasonable request.
